# RANKL neutralisation prevents osteoclast activation in a human *in vitro* ameloblastoma-bone model

**DOI:** 10.1177/20417314221140500

**Published:** 2022-12-24

**Authors:** Judith Pape, Deniz Bakkalci, Rawiya Al Hosni, Benjamin S Simpson, Kristiina Heikinheimo, Stefano Fedele, Umber Cheema

**Affiliations:** 1 UCL Centre for 3D Models of Health and Disease, Division of Surgery and Interventional Science, University College London, London, UK; 2 Research Department of Targeted Intervention, Division of Surgery and Interventional Science, University College London, London, UK; 3 Department of Oral and Maxillofacial Surgery, Institute of Dentistry, University of Turku and Turku University Hospital, Turku, Finland; 4 Eastman Dental Institute, Oral Medicine Unit, University College London, London, UK

**Keywords:** Odontogenic, neoplasm, RANK ligand, osteoblasts, osteoclastogenesis

## Abstract

Ameloblastoma is a benign, locally invasive epithelial odontogenic neoplasm of the jaw. Treatment of choice is jaw resection, often resulting in significant morbidity. The aim of this study was to recapitulate ameloblastoma in a completely humanised 3D disease model containing ameloblastoma cells, osteoblasts and activated osteoclasts to investigate the RANKL pathway within the ameloblastoma stromal environment and its response to the RANKL antibody denosumab. In vitro bone was engineered by culturing human osteoblasts (hOB) in a biomimetic, dense collagen type I matrix, resulting in extensive mineral deposits by day 21 forming alizarin red positive bone like nodules throughout the 3D model. Activated TRAP + human osteoclasts were confirmed through the differentiation of human CD14+ monocytes after 10 days within the model. Lastly, the ameloblastoma cell lines AM-1 and AM-3 were incorporated into the 3D model. RANKL release was validated through TACE/ADAM17 activation chemically or through hOB co-culture. Denosumab treatment resulted in decreased osteoclast activation in the presence of hOB and ameloblastoma cells. These findings stress the importance of accurately modelling tumour and stromal populations as a preclinical testing platform.

## Introduction

Ameloblastoma is a rare, benign intraosseous progressively growing epithelial odontogenic neoplasm characterised by expansion and a tendency for local recurrence if not adequately removed.^1^ Peak incidence is the fourth and fifth decade and the predilection site is the posterior region of mandible. Although a benign tumour, ameloblastoma behaviour is unpredictable and should be treated by resection and followed up for decades.^2^ One of the major pathways involved in the disease is the nuclear factor kB ligand or RANK ligand (RANKL) signalling pathway.^3^ RANKL is key coordinator in bone homoeostasis.^4^ In healthy bone stroma, osteoblasts lay down mineral and consistently release RANKL.^5^ Release of RANKL activates osteoclasts to carry out bone resorption.^6^ Osteocytes, former osteoblasts, form within the bone mineral and express RANKL actively for osteoclast formation and recruitment^7^ leading to the upholding of bone structure.^8^ Osteoprotegerin (OPG) maintains the balance between bone formation and excessive bone resorption.^9^ OPG competitively binds to RANKL and therefore stops its binding to osteoclasts and prevents their activation.^10^ It is hypothesised that ameloblastoma cells release RANKL themselves to stimulate excessive bone resorption by osteoclasts.^11^ Furthermore, ameloblastoma may create a positive feedback loop with osteoblasts to release more RANKL together. This will then lead to an abnormal amount of bone resorption, creating an empty cavity that the ameloblastoma tumour can grow into.^12^ The RANKL antibody denosumab has been used efficiently in giant tumour cells to inhibit osteoclast activation *in vitro* .^13^ Since the RANKL pathway may be significantly involved in ameloblastoma invasion, drugs neutralising this ligand are promising candidates to be used for treatment. In order to accurately recapitulate the ameloblastoma tumour stroma, several factors need to be considered. High biomimicry can be obtained by using adequate collagen concentrations (⩾10%) within a tissue-engineered 3D model utilising type I collagen.^14^ These types of models allow cell types such as epithelial tumour and corresponding stromal mesenchymal cells to function as if within their innate environment. In this study, the aim was to engineer a fully human 3D model of ameloblastoma incorporating bone forming osteoblasts and bone resorbing osteoclasts. Furthermore, this novel 3D model was to be used as a drug testing platform. The RANKL antibody denosumab was used to investigate the effect on osteoclasts activated in the presence of ameloblastoma cells. The hypothesis was that denosumab will decrease osteoclast activation thus leading to better disease outcome clinically.

## Methods

### Cell culture

AM-1 cells were kindly gifted by Prof. Harada from the Harada Group, Osaka University, Japan and grown in keratinocyte serum free medium (SFM). AM-3 cells were kindly gifted by Prof. Kishida and colleagues from Kagoshima University, Japan and grown in defined keratinocyte SFM. Both were supplemented with supplement mix, 10% foetal bovine serum (FBS) and 100 units/mL of penicillin and 100 µg/mL streptomycin. Human osteoblasts (hOB) isolated from femoral trabecular bone tissue (hip or knee joint region) were purchased through Promocell^®^ (Heidelberg, Germany) and cultured in osteoblast growth medium supplemented with supplement mix, 10% foetal bovine serum (FBS) and 100 units/mL of penicillin and 100 µg/mL streptomycin.

### Mineralisation assay

For the hOB cells to mineralise, cells were cultured in osteoblast mineralisation medium for a minimum of 21 days (Promocell, Heidelberg, Germany). The medium was supplemented with supplement mix, 10% foetal bovine serum (FBS) and 100 units/mL of penicillin and 100 µg/mL streptomycin (all Gibco™ through Thermo Fisher Scientific, Loughborough, UK). For 2D, 1 × 10^5^ cells were seeded per six-well plate. For 3D, 7 × 10^4^ cells were seeded into a 24-well dense collagen gel (see 3D model fabrication). The mineralisation medium was changed 100% for 2D every 48 h (h) and 50% for 3D every 48 h. 3D samples release several essential growth factors into the medium over time and thus a 50% media change would prevent the removal of these. Mineral deposits visible by eye from day 21. Cultures were kept for a maximum of 40 days (d).

### Human CD14+ monocyte differentiation into activated osteoclasts

Human CD14+ monocytes (hMoCD14+-PB) isolated from single donor peripheral blood were purchased through Promocell^®^ (Heidelberg, Germany). Cells were thawed according to manufacturer’s instructions and left for 24 h initially in a T25 flask undisturbed. After this, cells were spun at 240 G and counted. Yielding viable hMoCD14+-PB cells were incorporated and differentiated at 2 × 10^4^ cells per 24-well size dense collagen gel. Stimulation was performed using 50 ng/mL RANKL and 25 ng/mL MCS-F (both R&D Systems^®^ through Bio-Techne Ltd^®^ Abingdon, UK) in α-MEM medium supplemented with 25 mM HEPES for 10 days (Gibco™ through Thermo Fisher Scientific, Loughborough, UK). Media change was performed every 48 h.

### 3D model fabrication

**Figure 1.**
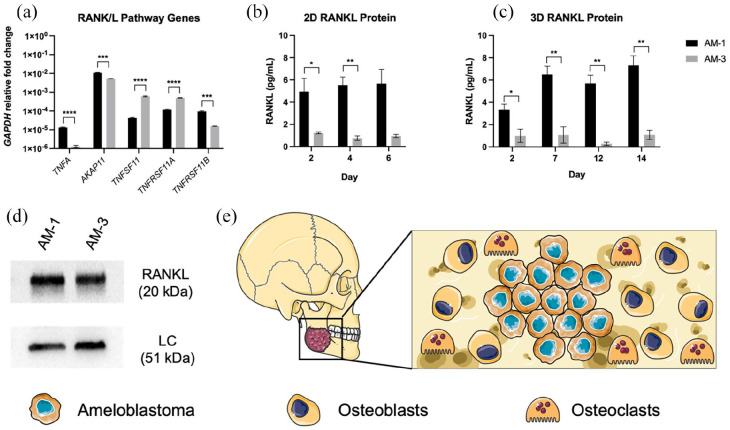
Characterisation of RANKL expression in ameloblastoma cell lines AM-1 and AM-3. (a) mRNA expression tumour necrosis factor alpha (TNFA), A-kinase anchor protein 11 (AKAP11), RANK ligand (TNFSF11), RANK ligand receptor (TNFRSF11A) and osteoprotegerin (TNFRSF11B). Fold change relative to glyceraldehyde-3-phosphate dehydrogenase (GAPDH) mRNA levels. (b) RANKL protein expression into medium within AM-1 and AM-3 2D monolayers and (c) 3D artificial tumour masses. (d) Western blots of RANKL protein for AM-1 and AM-3 2D monolayers with loading control beta-actin. All data represented as mean ± SEM. *p*-values representing *<0.05, **<0.005, ***<0.0005 and ****<0.00001. Schematic of the 3D model set up. (e) Schematic of the active ameloblastoma stromal environment involved in the RANKL pathway. This schematic was created using Created using Servier Medical Art according to the Creative Commons Attribution 3.0 Unported Licence guidelines 3.0 (https://creativecommons.org/s/by/3.0/, accessed on 29th June 2022).

3D constructs were fabricated as previously described.^15^ In summary, monomeric collagen type I of rat tail origin (First Link, Birmingham, UK) was mixed with 10X MEM (Gibco™ through Thermo Fisher Scientific, Loughborough, UK) and neutralising agent (N.A.) according to the RAFT™ protocol. After cellular addition, cross-linking is performed by adding 240 µL or 1.3 mL of cellular collagen mix into a 96-well or 24-well plate respectively, followed by incubation at 37°C for 15 min. Plastic compression is performed using RAFT™ absorbers (Lonza, Slough, UK) for 15 min resulting in dense 10% collagen constructs.^14^ In order to adhere different layers of dense collagen gels producing multicellular constructs as described in Figure 1 and Table 1, a drop of collagen mix was used.

**Table 1.** Cell types and sources for the 3D model set up.

Cell type and source (all human)	Differentiation and culture method	Mimicking structure
CD14+ monocytes (hMoCD14+-PB) by Promocell^®^	2 × 10^4^ cells/sample	Activated osteoclasts
	10% collagen	
	Day 10 differentiation	
Ameloblastoma cell lines AM-1 and AM-3 (Gifted)	5 × 10^4^ cells/sample	Ameloblastoma tumour
	10% collagen	
	Day 7 growth	
Osteoblasts (hOB) by Promocell^®^	7 × 10^4^ cells/sample	Mineralised bone
	10% collagen	
	Day 21 mineralisation	

### PMA, ionomycin and LPS treatment and hOB co-culture for RANKL release

To release RANKL, a chemical assay was used as previously described.^16^ In short, 50 ng/mL PMA, 1 µg/mL ionomycin and 10 µg/mL LPS extracted from *E. coli* (all through Sigma-Aldrich, Dorset, UK) were used to activate ADAM17/TACE, which is a RANKL ‘sheddase’. The treatment was left on AM-1 and AM-3 cells for 5 h and RNA samples were taken at 6, 24 and 48 h. Additionally, cells were stained for immunofluorescence at 48 h. In order to demonstrate whether RANKL production would be upregulated in a co-culture with hOB, a 1:1 seeding density was used and left for 48 h.

### Denosumab RANKL neutralisation

Denosumab biosimilar ICH4019 was purchased through IchorBio (Wantage, UK). Initially, a proliferation assay was performed on AM-1, AM-3 and hOB cells in 2D to assess effect on cell viability. For this, 4 × 10^3^ cells/well were seeded in a 96-well plate and left to attach for 24 h. Cells were treated twice at 72 h intervals with increasing concentrations (15, 30, 60 and 120 µg/mL). Cell viability was assessed with PrestoBlue^®^. For the multicellular 3D samples, constructs were treated twice at 72 h intervals using a concentration of 30 µg/mL as previously described.^13^

### Immunofluorescent staining

Cells were fixed with 10% neutrally buffered formalin (N.B.F.). Samples were permeabilised using 0.2% Triton X-100 and 1% bovine serum albumin (BSA) for 1 h (both Sigma-Aldrich, Dorset, UK). 1° (1:200) antibody incubation overnight at 4°C using RANKL (ab45039) or SPARC antibody (ab225716). 2° antibody (1:1000) was incubated for 2.5 h at room temperature with anti-mouse Alexa Fluor™ 488 IgG H&L (ab150113) or anti-rabbit DyLight^®^ 594 (ab96885) (all Abcam, Cambridge, UK). For phalloidin staining, samples were incubated for 30 min with Alexa Fluor™ 568 Phalloidin Kit (Invitrogen™ through Thermo Fisher Scientific, Loughborough, UK). All samples were counterstained with 4′,6-diamidino-2-phenylindole (DAPI) using NucBlue™ (Invitrogen™ through Thermo Fisher Scientific, Loughborough, UK). All fluorescent images were taken on the Zeiss AxioObserver using Zeiss ZEN software (Zeiss, Oberkochen, Germany).

### TRAP staining of human osteoclast in 3D collagen constructs

Activated osteoclasts were visualised using the B-Bridge International, Inc. TRAP Staining Kit (through 2BScientific, Heyford, UK). 3D samples were washed with PBS once and fixed with 10% N.B.F. for 30 min. Samples were washed three times with dH_2_O. Chromogenic substrate was added to the sample and incubated for 60 min at 37°C. This was followed by three 5 min washes with dH_2_O. All colourimetric images were taken on the Zeiss AxioObserver using Zeiss ZEN software (Zeiss, Oberkochen, Germany) and analysed using the Fiji ImageJ software.^17^

### Histology, alizarin red staining and BRAF V600E mutation specific immunohistochemistry

Samples were fixed in 10% N.B.F. overnight before routine processing. Samples were embedded horizontally into paraffin wax. Sections were 4 µm and mounted on Superfrost™ positively charged slides (VWR International, Poole, UK). Slides were rehydrated and Alizarin Red Solution (Millipore^®^ through Sigma-Aldrich, Dorset, UK) was applied to sections for 2 min, before being blotted and dehydrated in acetone for 10–20 s. This was followed by acetone-xylene (50:50) for 10–20 s. Slides were added to xylene for 5 min twice. Finally, samples were dehydrated. Images were taken on the Zeiss AxioObserver using Zeiss ZEN software (Zeiss, Oberkochen, Germany). Immunohistochemistry staining was performed by UCL IQPath histology services on the VENTANA BenchMark ULTRA instrument using the VENTANA OptiView DAB IHC Detection Kit and VENTANA ULTRA CC1 pre-treatment (all through Roche Diagnostics, Basel, Switzerland). BRAF V600E VE1 antibody (ab228461) was used at a 1:50 dilution with 28 min incubation (Abcam, Cambridge, UK).

### RNA extraction, cDNA transcription and qPCR

Samples were lysed using TRI Reagent™ and RNA was extracted using the TRIzol-Chloroform phase separation method^18^ (both Sigma-Aldrich, Dorset, UK). Complementary DNA (cDNA) was transcribed using the High-Capacity cDNA Reverse Transcription Kit (Applied Biosystems™ through Thermo Fisher Scientific, Loughborough, UK). Primers were designed according to the MIQE guidelines.^19^ Sequences can be found in Supplemental Table 1. All primers were ordered through Eurofins Genomics (Ebersberg, Germany) and used at concentration of 0.2 µM. qPCR was performed on the CFX96™ Touch System utilising the iTaq™ Universal SYBR™ Green Supermix (both Bio-Rad, Watford, UK). Relative gene expression was calculated using the ΔCt method,^20^ normalised to *glyceraldehyde-3-phosphate dehydrogenase (GAPDH)* expression with primers from literature.^21^

### Protein purification and western blotting

10 μg of protein was loaded and run at 200 V (Volts) for 45 min. Membranes were blocked for 1 h with 5% milk (Sigma-Aldrich, Dorset, UK) (in tris- buffered saline and 1% Tween 20 (TBST), both Bio-Rad, Watford, UK), and incubated with 1° antibody (1:200) for RANKL (ab45039) and (1:10,000) loading control beta-actin (ab8227) in 5% milk overnight at 4°C (both Abcam, Cambridge, UK). 2° antibodies (1:1000) IgG-HRP (sc-2314) (Santa Cruz Biotechnology, Dallas, USA) and P0448 (Dako through Agilent, Santa Clara, USA) were incubated for 1 h in 3% milk and visualised using Pierce™ ECL Western Blotting Substrate (Thermo Fisher Scientific, Loughborough, UK). Blots were imaged using the ChemiDoc™ XRS imaging system and Image Lab™ software (Bio-Rad, Watford, UK).

### Rankl Elisa

Media samples were taken from cells at longitudinal timepoints, spun down and stored at −80°C. To quantify active RANKL protein released into the media, the human TRANCE/RANK L/TNFSF11 DuoSet ELISA kit was used in conjunction with the DuoSet ELISA Ancillary Reagent Kit 2 (both by R&D Systems^®^ through Bio-Techne Ltd^®^ Abingdon, UK).

### Statistical analysis

All images were analysed using the Fiji ImageJ software.^17^ Graphs were created using GraphPad Prism 9 software. Data sets were tested for normal distribution using Shapiro-Wilk test (*n* = 3–7) or D’Agostino test (*n* ⩾ 8). Parametric tests: unpaired t test and one-way ANOVA with Dunnet’s post hoc correction and non-parametric tests: Mann-Whitney or Kruskal-Wallis test with Dunn’s multiple comparison correction. Statistical significance was *p* < 0.05, all data points are mean with standard error mean (SEM) with all experiments being *n* = 3 with 3–4 technical replicates.

## Results

### AM-1 and AM-3 ameloblastoma cell line gene expression and 3D proliferation

The AM-1 and AM-3 cell lines express several genes involved within the RANK ligand pathway (Figure 1(a)). The AM-1 cell line expresses significantly higher levels of *TNF-*α (*p* < 0.0001), *AKAP11* (*p* = 0.0003) and *TNFRSF11B* (*p* = 0.0002). Meanwhile, the AM-3 cell line expresses significantly higher levels of *TNFSF11* and *TNFRSF11A* (*p* =< 0.0001 for both). Levels of functional RANKL protein released into the cell medium in 2D (Figure 1(b)) and 3D (Figure 1(c)) over time. AM-1 cells expressed significantly higher levels of RANKL protein into the medium over time in 2D (*p* = 0.0363 and 0.0031 for day 2 and 4 respectively) and 3D (*p* = 0.0412, 0.0067, 0.0020 and 0.0029 for day 2, 7, 12 and 14 respectively. The amount of total RANKL protein was visualised through western blotting. As seen in Figure 1(d), both cell lines have high expression of total RANKL protein. Figure 1(e) is a schematic demonstrating the interplay between ameloblastoma cells, osteoblasts and osteoclasts within the active bone stroma.

### Ameloblastoma cell lines can be triggered to shed RANKL chemically or in co-culture with human osteoblasts

**Figure 2.**
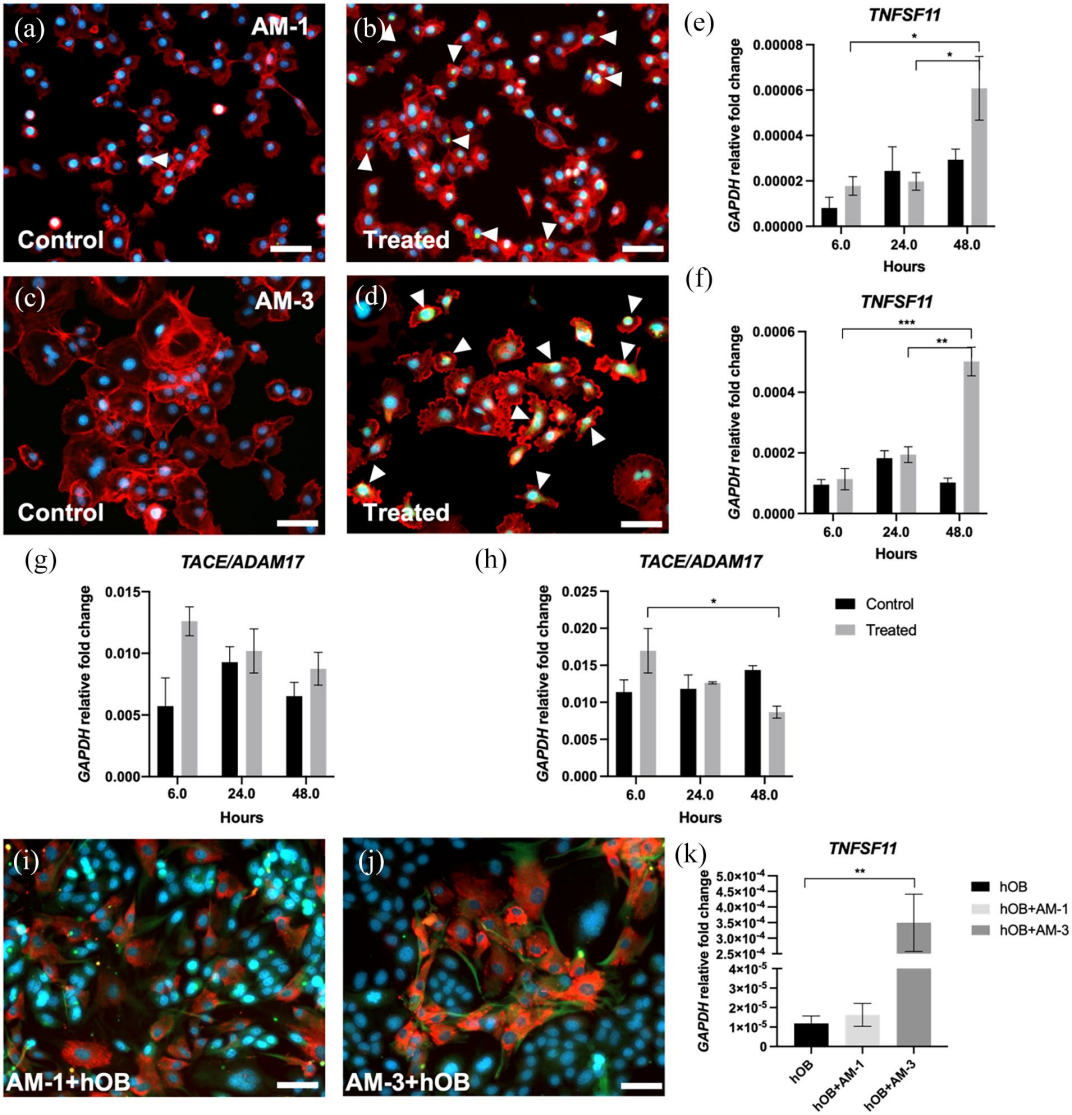
Triggering RANKL release in AM-1 and AM-3 cells. (a) Control and (b) treated AM-1 cells. (c) Control and (d) treated AM-3 cells. Red = Phalloidin, green = RANKL, blue = DAPI and scale bar = 50 µm. (e) mRNA expression of TNFSF11 (RANKL) in AM-1 and (f) AM-3 cells after treatment. (g) mRNA expression of A disintegrin and metalloprotease 17 (TACE/ADAM17) in AM-1 and (h) AM-3 cells after treatment. (i) Co-culture of AM-1 and hOB cells. (j) Co-culture of AM-3 and hOB cells. Red = Osteonectin (SPARC), green = RANKL, blue = DAPI and scale bar = 50 µm. (k) RANKL mRNA expression of hOB cells and in co-culture with AM-1 or AM-3 cells. All data represented as mean ± SEM with fold change relative to glyceraldehyde-3-phosphate dehydrogenase (GAPDH) mRNA levels. *p*-values representing *<0.05, **<0.005 and ***<0.0005.

To release membrane bound RANKL protein from the ameloblastoma cell lines and to increase RANKL production, PMA, ionomycin and LPS treatment was applied. Figure 2(a) to (d) show increased staining for RANKL upon treatment for AM-1 and AM-3 cells as indicated by the white focus arrows. The morphology of AM-3 cells (Figure 2(d)) was affected. Figure 2(e) and (f) demonstrate significant upregulation of *RANKL* mRNA upon treatment over time. The levels increased significantly from 6 to 48 h (*p* = 0.0306) and from 24 to 48 h (*p* = 0.0370) in the treated AM-1 group. This trend was also observed in the AM-3 treated group, as *RANKL* levels increased significantly between 6 and 48 h (*p* = 0.0008) and between 24 and 48 h (*p* = 0.0027). Figure 2(g) and (h) show an initial upregulation of *TACE* mRNA upon treatment at 6 h, which shows a decreasing trend over time. The decrease within the AM-3 treated group was significant between 6 and 48 h (*p* = 0.0393). Figure 2(i) and (j) show increased staining in AM-1 and AM-3 cells when in co-culture with hOB for 48 h. hOB cells also demonstrate increased staining for RANKL. *RANKL* mRNA levels were upregulated significantly in AM-3/osteoblast co-cultures only (*p* = 0.0075), shown in Figure 2(k).

### Human osteoblasts mineralise in dense collagen constructs

**Figure 3.**
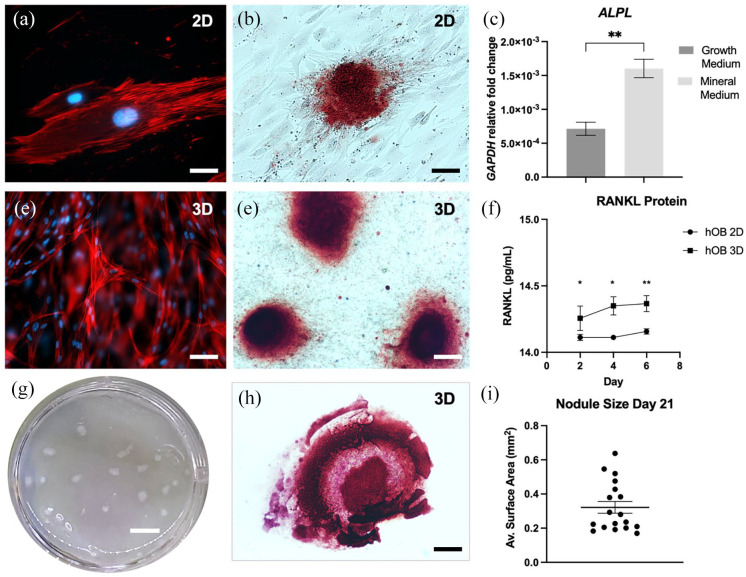
Tissue-engineering human bone in 3D. (a) Human osteoblast (hOB) cells grown in 2D. (b) Alizarin red staining of mineralisation in 2D after 21 days. (c) mRNA expression of alkaline phosphatase (ALPL) in hOB when grown in normal or mineralisation media for 21 days. Fold change relative to glyceraldehyde-3-phosphate dehydrogenase (GAPDH) mRNA levels. (d) hOBs grown in 3D. (e) alizarin red staining of hOB cells grown in 3D. (f) RANKL protein expression of hOB into cell medium in 2D versus 3D. (g) Bone nodule formation by hOB in 3D after 21 days in mineralisation medium and (h) respective alizarin red staining within sectioned nodule. (i) Average nodule size by day 21. For all images red = phalloidin and blue = DAPI. Scale bar = 25 µm for (a) 100 µm for (b) 50 µm for (d) 500 µm for (e and h) and 2 mm for (g) Data represented as mean ± SEM. *p*-values representing *<0.05, **<0.005 and ***<0.0005.

Human osteoblasts (hOB) had an elongated and flat morphology in 2D (Figure 3(a)) and mineralised after 21 days, seen through alizarin red staining in Figure 3(b). In Figure 3(c), the mineralisation gene *alkaline phosphatase (ALPL*) was significantly upregulated in mineralised culture, compared to cells grown in normal medium (*p* = 0.0059). When grown in dense collagen, the hOB cells obtained a 3D orientation (Figure 3(d)). The cells formed mineralised, alizarin red positive ‘bone nodules’ after 21 days (Figure 3(e)). RANKL protein release into the media was significantly higher over time by the hOB when grown in 3D as compared to 2D (*p* = 0.0390, 0.0346 and 0.043 for day 2, 4 and 6 respectively) as seen in Figure 3(f). The visible nodules were (Figure 3(g)) sectioned and stained for alizarin red showing hollow centres (Figure 3(h)). The bone nodules were quantified for surface area (Figure 3(i)) with mean values at 0.322 ± 0.146 mm^2^ (mean ± STDEV).

### Denosumab inhibits human osteoclast activation in co-culture

**Figure 4.**
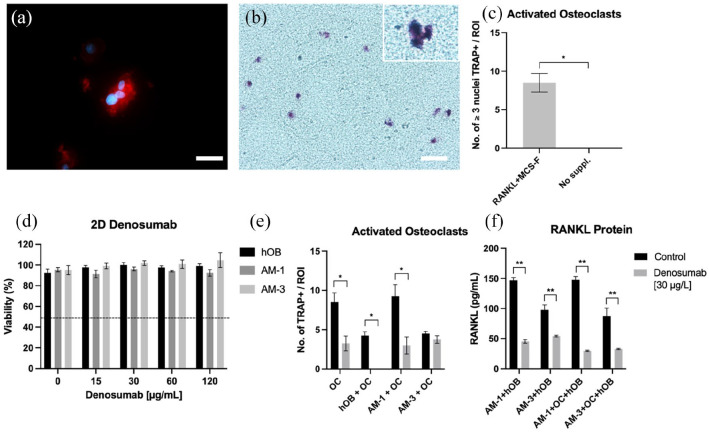
Denosumab effect on AM, hOB and activated osteoclast formation. (a) (J) Multinucleated, (⩾3 nuclei/colony) activated osteoclast formation in 3D with (b) respective TRAP staining. (c) Quantification of activated osteoclasts per ROI at 10x magnification compared to undifferentiated CD14+ monocytes. (d) Cell Viability of hOB, AM-1 and AM-3 cells after treatment with varying denosumab concentrations. (e) Number of TRAP positive colonies after denosumab treatment in different 3D co-cultures. (f) RANKL protein expression after denosumab treatment in different 3D co-cultures. For (a) red = phalloidin and blue = DAPI. Scale bar = 50 µm for (a) and 25 µm for (b). Data represented as mean ± SEM. *p*-values representing *<0.05, **<0.005 and ***<0.0005.

Activated human osteoclast populations were derived by differentiating CD14+ monocytes from human peripheral blood within dense collagen constructs. As seen in Figure 4(a), CD14+ monocytes started forming multinucleated (⩾3 nuclei) cell clusters by day 10 of differentiation. Figure 4(b) demonstrates positive TRAP (TRAP+) staining of these colonies, confirming activated osteoclast status. There was an average of 8.5 ± 2.38 colonies forming per ROI on a 10x magnification. Figure 4(d) confirms that cellular viability was not affected by increasing denosumab concentrations within the different cell populations. Figure 4(e) shows TRAP+ colonies were significantly decreased when treated with denosumab in monoculture (*p* = 0.0136) and when in co-culture with hOB and AM-1 cells (*p* = 0.0286 and 0.0146). When in the presence of hOB only, denosumab completely blocked the formation of TRAP+ colonies. AM-3 cells blocked denosumab action and the number of TRAP+ colonies was not decreased. Figure 4(f) shows the amount of RANKL released into cell medium. In the co-culture of AM-1 + hOB, RANKL protein was significantly reduced in the denosumab treated group (*p* < 0.0001). Similarly, within the AM-3 + hOB group (*p* = 0.0087). In tri-culture (AM + hOB + OC) RANKL protein was significantly decreased due to denosumab treatment in the presence of AM-1(*p* < 0.0001) and AM-3 cells (*p* = 0.0018) group.

## Discussion

The development of 3D, biomimetic and physiologically relevant tissue models is critical as accurate tissue mimics. By culturing osteoblasts and activated osteoclasts within dense 3D collagen constructs, the actions of ameloblastoma cells were studied.

There are a limited number of studies using tissue-engineered 3D ameloblastoma models.^22^ Bakkalci et al.^23^ previously characterised AM-1 and AM-3 cells in relation to rat bone formation *in vitro* . The benefits of using a 3D spatial configuration are that the inherent pathological phenotype of tumour cells will be stimulated. This is especially critical for ameloblastoma, which is often described as having a plexiform or follicular appearance. Justification for biomimetic complexity and a clear role of the tumour-stroma interaction, was validated by RANKL protein release. Active RANKL protein expression by single cell cultures of AM-1 and AM-3 cells was low. This was attributed to the lack of interaction between ameloblastoma cells and the native stromal cells. Following the addition of human osteoblasts significant increases in released RANKL were observed. It has long been postulated that the interaction of ameloblastoma cells with resident osteoblast cells in bone results in pathological osteoclast activation.^11^ It is this osteoclast activation that is attributed to causing the bone loss associated with ameloblastoma. The importance of tumour-stroma interactions in terms of cell invasion and progression is well established.^24^ This interaction is not limited to tumour and stromal cells but is also relevant for the tumour and stroma matrix.^25^ In terms of tumours in bone or metastasis to bone, the critical balance of bone remodelling, by resident osteoblasts and activated osteoclasts remains key to bone homeostatsis.^26^ The majority of previous studies focused on how ameloblastoma interferes with osteoclastogenesis and induces bone resorption by upregulating factors such as RANKL.^11,27^ The upregulation of RANKL expression, and its subsequent reduction by the addition of denosumab in the model presented herein, demonstrates the biomimetic and responsive nature of this novel system. It is also worth considering why the actions of denosumab in AM-3 cultures resulted in reduced free RANKL expression, however, did not subsequently result in a decrease in the number of activated osteoclasts. This may possibly be due to RANKL independent pathways inducing osteoclastogenesis. Examples of this include pathways where IL-6 and IL-11 play a crucial role.^28^

Further elaboration of the 3D model showed clear formation of mineralised nodules by resident osteoblasts. Moreover, the addition of differentiated monocytes, allowed for an activation towards the osteoclast phenotype, evidenced by TRAP+ colonies. These colonies resembled osteoclast clusters observed in human bone. Whilst other cell populations are of pathological importance within the ameloblastoma tumour stroma, having established a humanised model with these cellular populations *in vitro* will have impact for other bone related fields of study. Future works including fibroblast populations and immune cell populations such as tumour associated macrophages would be of great value.^29^ Furthermore, incorporating endothelial cells such as HUVECs into the stromal compartment could aid in further investigating the intratumoral hypoxia observed in ameloblastoma.^30^ It would also be of great value to incorporate primary ameloblastoma cells into the model due to the limitations of immortalised cell lines and their homogenous nature. Exploration of the RANKL antibody action of denosumab clearly showed significant inhibition of osteoclast activation in the presence of osteoblasts and ameloblastoma cells. Previous work has ascertained that culturing monocytes in 3D collagen gels resulted in an similar differentiation towards osteoclasts under equivalent conditions with increased sensitivity to reduced concentrations of RANKL.^31^ Targetting RANKL signalling has been studied in other tumours as well as in bone metastasis. Denosumab was found to be well-tolerated in premenopausal early-stage breast cancer patients to prime luminal breast cancer for immunotherapy.^32^ In a clinical trial, denosumab improved overall survival of patients with non-small-cell lung carcinoma (NSCLC) adenocarcinomas and squamous tumours.^33^ Denosumab has not yet been tested in ameloblastoma and this model will help in understanding how denosumab interferes with ameloblastoma mediated osteoclast activation.

In conclusion, a completely humanised model containing osteoblasts and activated osteoclasts of human origin was established demonstrating mineralised nodules and TRAP+ colonies. Whilst investigating the RANKL pathway, it was found that denosumab could inhibit osteoclast activation to a significant extent in the presence of osteoblasts and ameloblastoma cells. This has led to a better understanding of ameloblastoma disease and may have clinical implications for the development of novel drug therapies.

## Supplemental Material

sj-docx-1-tej-10.1177_20417314221140500 – Supplemental material for RANKL neutralisation prevents osteoclast activation in a human in vitro ameloblastoma-bone model Click here for additional data file. Supplemental material, sj-docx-1-tej-10.1177_20417314221140500 for RANKL neutralisation prevents osteoclast activation in a human in vitro ameloblastoma-bone model by Judith Pape, Deniz Bakkalci, Rawiya Al Hosni, Benjamin S Simpson, Kristiina Heikinheimo, Stefano Fedele and Umber Cheema in Journal of Tissue Engineering
