# Supplementary material for: RANKL neutralisation prevents osteoclast activation in a human in vitro ameloblastoma-bone model
Source: J Tissue Eng. 2022 Dec 24;13:20417314221140500. doi: 10.1177/20417314221140500 (PMC9793035; doi:10.1177/20417314221140500)
Supplement: sj-docx-1-tej-10.1177_20417314221140500 – Supplemental material for RANKL neutralisation prevents osteoclast activation in a human in vitro ameloblastoma-bone model [file sj-docx-1-tej-10.1177_20417314221140500.docx]

**Supplementary Material**

*Supplementary Table 1: Primer sequences and amplicon size*

| **Gene** | **F’ Primer** | **R’ Primer** | **Product Size (bp)** |
| --- | --- | --- | --- |
| *TNF-α* | CTCTTCTGCCTGCTGCACTTTG | GGGTTTGCTACAACATGGGCTAC | 149 |
| *AKAP11* | TCTTTATTGCAGAGTCAGAAGGAAC | AAACCCAGAAATGTGACCTCAG | 101 |
| *TNFSF11* | CAGAGCAGAGAAAGCGATGGTG | TTATGGGAACCAGATGGGATGTC | 123 |
| *TNFRSF11A* | TGGGACGGTGCTGTAACAAATG | GGGCCTTGCCTGTATCACAAAC | 165 |
| *TNFRSF11B* | AAACGGCAACACAGCTCACAAG | GCTGCTCGAAGGTGAGGTTAGC | 163 |
| *TACE* | TGAAGGAAGGTGTCCAGTGCAG | GGCGGGCACTCACTGCTATTAC | 148 |
| *ALPL* | GATGGGATGGGTGTCTCCACAG | GGACCTGGGCATTGGTGTTG | 145 |
